# Scale-aware feature modeling and dynamic sampling enable arbitrary-scale image super-resolution

**DOI:** 10.1038/s41598-026-48417-2

**Published:** 2026-04-18

**Authors:** Qianqian Tang, Yuanyuan Zhou, Xing Liu, Liwa Ma, HaoXin Zheng, Yucong He, Tianwen Zhang

**Affiliations:** 1 The College of Chinese & ASEAN Arts of CDU, Sichuan, China; 2 The Sichuan Aerospace Electronic Equipment Research Institute, Sichuan, China; 3 Faculty of Geosciences and Environmental Engineering, Southwest Jiaotong University, Chengdu, China

**Keywords:** Engineering, Mathematics and computing

## Abstract

Single-Image Super-Resolution (SISR) has witnessed a significant shift from traditional methods to deep learning, leading to flourishing developments recently. However, effectively handling arbitrary scales (e.g., integer, non-integer, or asymmetric) with a single model remains a challenging task. Existing SISR models fail to adequately perceive scale variations during the extraction of resolution features for arbitrary scales, while their limited local receptive fields hinder the modeling of global image structures. Furthermore, due to insufficient consideration of the impact of scale factors and local feature diversity on the upsampling stage, the generation of non-integer scale super-resolved images often suffers from incoherent details or abrupt transitions. To address these limitations, we proposed a cross-scale dynamic arbitrary-scale super-resolution network (CDASSR-Net) from the perspectives of cross-scale dynamic feature extraction and adaptive upsampling. Firstly, we propose a scale-aware feature adaptation (SAFA) module that adaptively adjusts filters according to the scale factor. Meanwhile, the cross-scale feature fusion via skip connections is proposed to better accommodating the demand for multi-scale feature representation and mitigating the loss of detail information common in deep networks performing such fusion. Then, we design a locally-adaptive scale-aware upsampler (LASU) module, which dynamically generates filters based on the input scale information, enabling upsampling to generalize to arbitrary resolutions. Extensive experiments conducted on various benchmark datasets demonstrate that integrating the proposed modules into fixed-scale SR networks allows them to achieve satisfactory performance on non-integer or asymmetric scales, while maintaining superior performance on integer scales. The code is available at https://github.com/Zheng4x/CDASSR.

## Introduction

Image super-resolution reconstruction aims to recover a high-resolution image from a single low-resolution input. The resolution of a digital image fundamentally quantifies its pixel density, where higher resolution values correspond to greater information capacity. Consequently, single-image super-resolution (SISR) techniques have become a focal research area in numerous fields including remote sensing, medical imaging, and image compression and transmission^1^. However, complex acquisition environments and uncertain hardware infrastructure often impede high-resolution image capture. Furthermore, image transmission processes typically impose compression and downsampling operations to enhance efficiency, inevitably degrading the original image information. Therefore, reconstructing high-resolution images is crucial for improving visual quality and enabling downstream application services.

Compared to upgrading hardware systems, SISR offers a flexible and cost-effective alternative by employing specialized algorithms to restore high-resolution images from low-resolution inputs^2^. Nevertheless, super-resolution reconstruction is fundamentally ill-posed: under the underdetermined conditions of the observation model, no unique analytical solution can be derived through traditional algebraic methods. This inherent ambiguity makes achieving satisfactory reconstruction results highly challenging.

Traditional SISR methods primarily rely on mathematical modeling and handcrafted priors to approximate optimal estimates from infinitely many potential solutions^3–5^. However, they struggle to achieve a dynamic balance between structural fidelity and model stability, often involving computationally intensive optimization processes. Recently, advancements in deep learning have driven the evolution of super-resolution architectures from interpolation-based upscaling to learning-based upsampling^6,7^. Conventional Convolutional Neural Networks (CNNs) exhibit inherent limitations in modeling long-range dependencies, primarily due to the local receptive field constraints and static weight allocation characteristic of convolutional operations. These limitations result in generated images lacking global semantic coherence and hinder effective adaptation to contemporary multi-scale super-resolution tasks.

Most existing methods primarily target single-scale super-resolution at specific integer scale factors (e.g., 2×, 4×). In contrast, multi-scale super-resolution (MSSR) aims to achieve arbitrary image magnification using a single model, unrestricted to symmetric integer scales, representing a more generalized super-resolution paradigm. As an extension of single-scale SR, MSSR methods incorporate reconstruction algorithms capable of arbitrary upscaling while maintaining high quality. However, the majority of super-resolution approaches lack support for arbitrary-scale reconstruction within a single model. Magnifying images to different scales necessitates retraining multiple dedicated models, significantly increasing computational and storage overhead^8,9^.

Employing multiple branches within deep learning models to handle multi-scale tasks constitutes the most intuitive solution^10–12^. However, this approach essentially relies on post-training manual selection of integer scale factors, suffering from inherent limitations such as operational redundancy. The MetaSR framework^13^, based on meta-learning, pioneered the introduction of an end-to-end deep network for continuous-scale super-resolution, which employs fully-connected (FC) layers to dynamically predict upsampling weights for different scale factors during the upsampling phase. Nevertheless, its exclusive reliance on scale information prevents adaptation of backbone network features. Adaptive upsampling via dynamic convolution can parameterize filters and adaptively adjust filter weights based on input characteristics^14–16^. Yet, dynamic convolution’s dependence on local neighborhood information aggregation hinders the recovery of high-frequency textures linked to global semantics. Additionally, it remains susceptible to input noise or abrupt scale factor variations, often leading to kernel parameter oscillation or outlier generation. The core challenge for stable arbitrary-scale resolution lies in constructing a framework featuring scale-decoupled feature extraction and dynamic self-adaptive reconstruction^17,18^.

To mitigate the aforementioned issues, we proposed a cross-scale dynamic arbitrary-scale super-resolution network (CDASSR-Net) based on feature scale-awareness and dynamic sampling. Specifically, instead of relying solely on input scale factors for learning, we design an effective scale-aware feature adaptation (SAFA) module to adapt features to specific scale factors. Recognizing that the feature mapping function of traditional convolution lacks scale-equivariant properties^19^, the proposed SAFA module adaptively adjusts filter parameters based on the scale factor. Furthermore, acknowledging that traditional feature fusion methods lack efficiency and that deep networks are prone to losing detail information during cross-scale fusion, we introduce efficient cross-scale feature fusion via skip connections. This design better accommodates the requirements of multi-scale feature representation^20^. Finally, we enable the network to adapt to specific scale factors by dynamically generating convolutional filters conditioned on the input scale information, implemented via conditional convolution. Collectively, equipped with these designs, the proposed CDASSR-Net can effectively perceive and extract multi-scale features, achieving high-quality super-resolution for images at arbitrary scales.

The main contributions of this work are summarized as follows:

1. We introduce the arbitrary-scale super-resolution network, scale-aware feature adaptation (SAFA) and locally-adaptive scale-aware upsampler (LASU) modules. These components enable feature learning to dynamically adapt to diverse scale factors for SR reconstruction. Both modules are designed as plug-and-play components that can be readily integrated into most existing SR networks.
2. The SAFA module adaptively recalibrates feature weights based on input characteristics and the target scale factor, addressing limitations in scale and local structural adaptability. The LASU module dynamically generates convolutional filters conditioned on scale information, enabling arbitrary-scale reconstruction without structural artifacts.
3. Extensive experiments demonstrate the effectiveness of the proposed method, yielding significant improvements over baseline models. Our approach achieves state-of-the-art performance on both quantitative metrics and visual quality compared to existing arbitrary-scale SR methods.

## Related works

### Super-resolution problem statement

Image super-resolution is a classic ill-posed inverse problem^21^, and its observation model can be formulated as follows:

1 $$\:y=Hx$$

Here, $\:x$ represents the unknown high-resolution (HR) image, and $\:y$ denotes the observed low-resolution (LR) image. The matrix $\:H$ is the degradation operator of the imaging system^22^, typically encompassing blur, geometric transformations, and down-sampling operations. To reconstruct the approximate original HR image $\:x$, super-resolution (SR) techniques are employed to solve the aforementioned inverse problem. However, this inverse problem is intrinsically ill-posed, meaning that infinitely many high-resolution solutions satisfy the given constraints. Therefore, it is essential to incorporate appropriate prior knowledge to effectively constrain the solution space, thereby constructing a solvable SR problem model as follows:

2 $$\:\widehat{x}=argmin \left\| y-Hx \right\|+\lambda\:E\left(x\right)$$

Here, $\:E\left(x\right)$ represents the prior information regarding the high-resolution (HR) image. The first term in Eq. (2) measures the discrepancy between the reconstructed image and the observed image, while the second term encodes constraints on the reconstruction imposed by prior knowledge about the image. The parameter $\:\lambda\:$ is a regularization coefficient used to balance these two terms. The prior information can be embedded explicitly or implicitly into the modeling of HR images. Classic explicit regularization methods include Tikhonov regularization^23,24^ and total variation (TV) regularization^25,26^. Although such priors improve reconstruction stability to a certain extent, their generalization capability is often limited by the difficulty in precisely modeling the underlying distribution of HR images in advance. Recently, the advent of deep learning has provided a novel paradigm for SR tasks. Numerous SR methods have started employing data-driven prior modeling strategies, leveraging large-scale image datasets to learn the statistical distributions of image features, thus obtaining more expressive priors tailored for specific tasks.

### Single-scale super-resolution algorithm based on deep learning

**Fig. 1:**
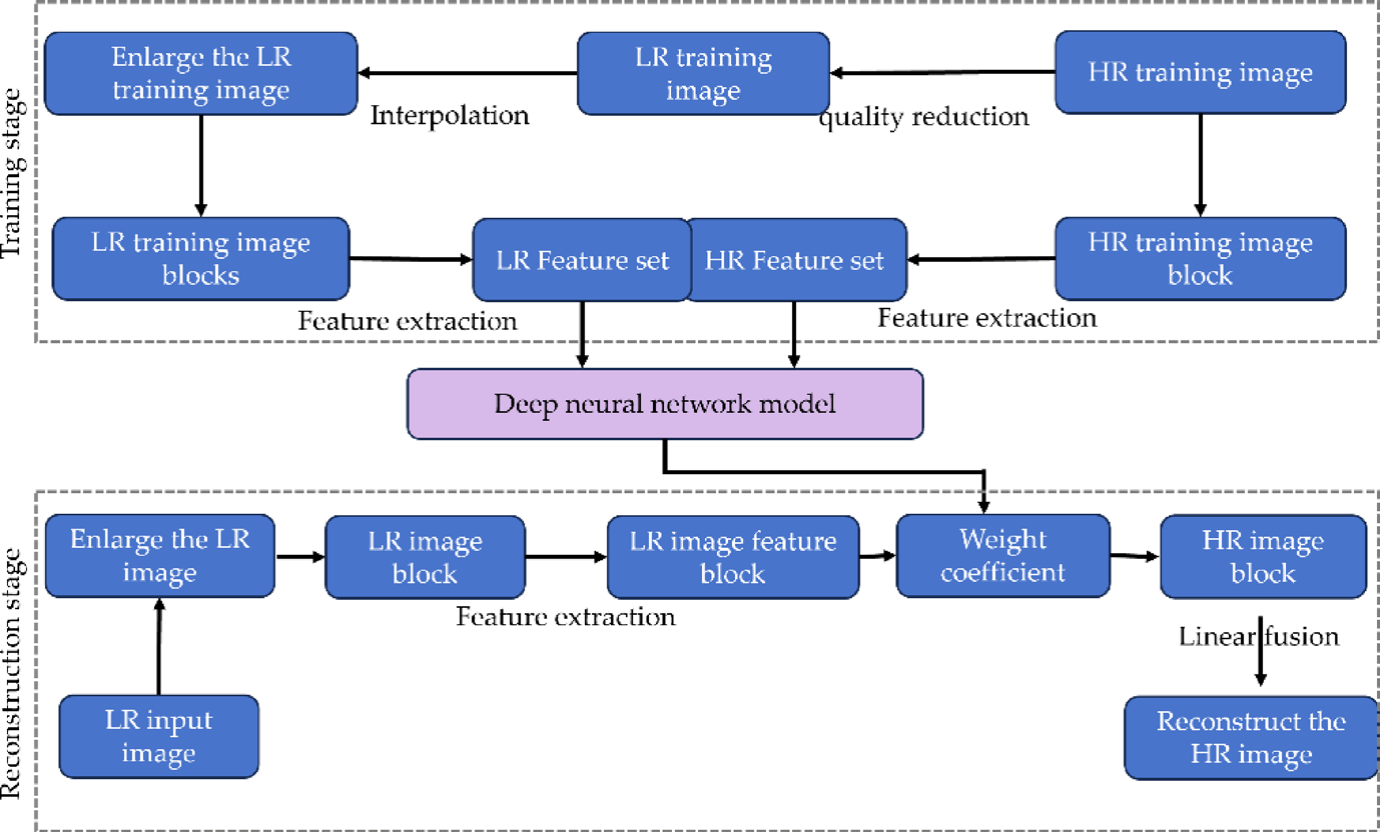
The general concept of deep learning-based super-resolution (SR) algorithms.

With the remarkable progress in deep learning, learning-based methods, benefiting from large-scale datasets and powerful neural network architectures^27^, have achieved increasingly challenging and impressive results in the field of image processing, drawing significant attention in recent years^28,29^. The general concept of deep learning-based super-resolution (SR) algorithms is illustrated in Fig. 1. The core idea involves generating pairs of low- and high-resolution images through synthetic degradation models as training data, then employing deep neural networks to learn the nonlinear mapping between them. Consequently, these methods can effectively recover lost high-frequency details, structural features, and texture information from low-resolution images, ultimately enabling high-quality, end-to-end image reconstruction^30,31^.

Early efforts in constructing super-resolution (SR) network models primarily focused on designing models with linear structures or deeper architectures employing a large number of convolutional kernels to achieve superior performance^32,33^. Dong et al.^34^ first introduced a three-layer convolutional network to the SR task; however, the interpolation operation involved led to the loss of image details and texture information. As an improvement, FSRCNN^35^ added a deconvolution layer after feature mapping to obtain the high-resolution image. Although this method could enhance image reconstruction, it was prone to generating blocking artifacts when reconstructing images at different magnification factors. One refinement approach involved adding a sub-pixel convolution layer at the network’s end^36^. Nevertheless, when processing texture-rich images, this could result in blurring or distortion, adversely affecting the quality of the reconstructed image. Lim et al. proposed a more performant residual network termed EDSR^37^. This network stacked deeper and wider residual blocks, introducing more parameters and a more complex structure to boost SR performance, but consequently led to an excessively large model size. In contrast, DRRN^38^, while also featuring a deep architecture, employed parameter sharing and repetitive structures across its modules, resulting in a relatively low parameter count. Furthermore, this model utilized a multi-path local residual structure alongside a global residual structure, which helped alleviate training difficulties and better preserve image details and texture information. However, these methods incurred substantial computational overhead during non-local operations. Additionally, constrained by the inherent local bias of CNNs, they failed to capture crucial long-range dependencies, ultimately reaching a performance plateau.

### Arbitrary scale super-resolution algorithm based on deep learning

In contrast to single-scale super-resolution (SR) methods, arbitrary-scale super-resolution aims to achieve magnification at arbitrary scales using a single model. This capability has broad applications across various fields, including satellite remote sensing, medical imaging, and security surveillance^39–43^. However, research on arbitrary-scale video super-resolution (VSR) remains scarce until recently. Meta-SR pioneered the use of a single model for arbitrary fractional-scale SR without interpolating arbitrary scale factors. Meta-SRAN replaced the feature extraction module of Meta-SR with a second-order attention network (SAN)^44^ for medical image SR. The aforementioned studies can be categorized as symmetric scale factor methods, whereas asymmetric scale factor methods support SR processing for low-resolution (LR) images with unequal aspect ratios. Wang et al. developed the Arbitrary-scale Super-Resolution (ArbSR) network^45^, which employs an upsampling module composed of scale-aware upsampling layers. By utilizing conditional convolution within the plug-in module to generate dynamic scale-aware filters, ArbSR adapts to arbitrary scale factors. Wang et al. proposed a Binary Lightweight SR (BLiSR) method^46^ to reduce computational and memory burdens and enhance SR practicality. Beyond incorporating scale information in the upsampling module, the Residual Scale Attention Network (RSAN) for arbitrary-scale image SR (ISR)^47^ introduced a scale attention module designed to learn discriminative features of LR images by incorporating the scale factor as prior knowledge^48^. Dynamic convolution was adopted to achieve adaptive upsampling, and the correlation between SR tasks at different scales was validated from the perspectives of degradation kernels and filters. To fully leverage content information, the weights of the upsampling convolution kernels should be both position-dependent and content-dependent. Although existing algorithms demonstrate promising reconstruction performance, most network models fail to effectively perceive scale variations during the extraction of arbitrary-scale resolution features. Furthermore, constrained by their local receptive fields, they struggle to model the global image structure. Additionally, when handling non-integer scaling factors (e.g., 2.1×, 3.7×), the generation of high-frequency details (e.g., edges, textures) often results in visually incoherent or abrupt transitions.

This paper addresses the limitations of existing network models in effectively perceiving scale variations during feature extraction for arbitrary-scale resolution and their difficulty in modeling global image structures due to local receptive fields. We propose a scale-aware feature adaptation module to enable the backbone network encoder to extract image features from different scales and regions. Simultaneously, multi-level feature propagation is achieved via skip connections. To tackle the problem of visually incoherent or abrupt generation of high-frequency details (e.g., edges, textures) when processing non-integer scaling factors (e.g., 2.1×, 3.7×), we propose a locally-adaptive scale-aware upsampler module designed for arbitrary-scale image super-resolution.

It is worth noting that our proposed locally-adaptive scale-aware upsampler (LASU) differs fundamentally from the dynamic convolution–based approach in Zhang et al.^43^. While the method in Ref^43^. generates dynamic convolution kernels for each input feature map at fixed scales, LASU is explicitly designed for arbitrary-scale super-resolution. First, LASU conditions the upsampling weights on the target scale factor, enabling continuous-scale reconstruction. Second, it dynamically samples features according to the continuous scale, reducing computation and allowing a single model to handle multiple scales. Finally, LASU integrates multi-level features from different encoder stages to improve reconstruction quality at arbitrary scales.

## Method

### The framework of the network

**Fig. 2:**
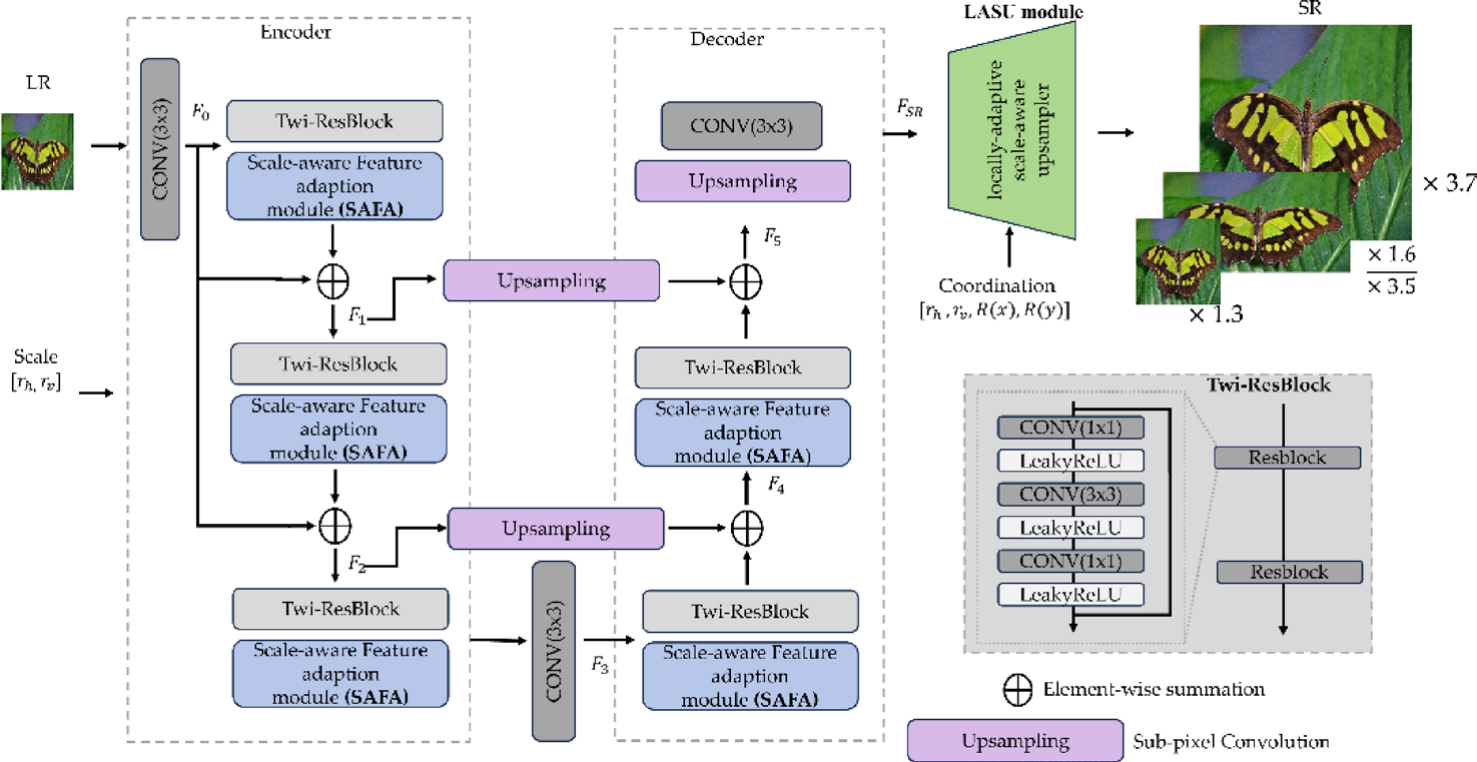
The framework of the CDASSR-Net. LR image from DIV2K dataset 0829.

The overall network structure of CDASSR-Net is shown in Fig. 2. The overall architecture consists of three components: the encoder, the decoder, and the LASU module.

For an input low-resolution (LR) image $\:{L}_{LR}$, the encoder first transforms it into a latent feature space. The upsampling module is critical for achieving super-resolution (SR) at arbitrary resolutions, while the decoder, in conjunction with the LASU module, maps the super-resolved latent representation back to the image domain at an arbitrary scale, yielding the high-resolution output image $\:{L}_{SR}$. The objective of the CDASSR-Net is to make $\:{L}_{SR}$ approximate the ground truth high-resolution image $\:{L}_{HR}$ as closely as possible. Initially, $\:{L}_{LR}$ is processed by a shallow feature extraction layer to obtain the base feature representation $\:{F}_{0}$:

3 $$\:{F}_{0}=\:Conv\left({L}_{LR}\right)$$

Here, $\:Conv$ denotes a 3 × 3 convolutional layer for initial feature extraction. Subsequently, a series of Twi-ResBlocks and Feature Adaptation Blocks (FABs) are employed to extract coarse-to-fine deep features from the image. The inputs to this stage are the shallow features $\:{F}_{0}$ and the two scale factors $\:{r}_{h}$ (horizontal) and $\:{r}_{v}$ (vertical), representing the scaling information. As illustrated in Fig. 2, the Twi-ResBlock structure comprises two cascaded residual networks (ResNets), designed to strike a balance between efficiency and accuracy in multi-scale feature extraction. Notably, the Twi-ResBlock can be substituted with more advanced backbones, such as ConvNeXt or Transformer architectures. The detailed architecture of the Feature Adaptation Block will be introduced in the subsequent section. Consequently, a sequence of latent features $\:{F}_{2}$, $\:{F}_{3}$, … is obtained during the encoding phase, as follows:

4 $$\:{F}_{1}={f}_{rf}\left({F}_{0}\right)+{F}_{0}$$

5 $$\:{F}_{2}={f}_{rf}\left({F}_{1}\right)+{F}_{0}$$

6 $$\:{F}_{3}=\:Conv\left({f}_{rf}\right({F}_{2}\left)\right)$$

where $\:{f}_{rf}(\cdot\:)$ represents the function composed of the cascaded Twi-ResBlocks and Feature Adaptation Block. To address the issues of information loss and insufficient global understanding inherent in the unidirectional information flow of traditional cascade networks, we employ dense cross-level feature Fusion. This mechanism ensures that the high-frequency details (e.g., textures, edges) crucial for reconstruction are not diluted or lost during the decoding process. The features obtained during the decoding phase can then be expressed as:

7 $$\:{L}_{SR}={f}_{s}({F}_{SR},{r}_{h},{r}_{v})$$

where $\:{f}_{s}(\cdot\:)$ denotes the reconstruction process performed by the Scale-Aware Upsampling Module Regarding model training optimization, we adopt the L1 loss as the network’s objective function, defined as follows:

8 $$\:L\left(\alpha\:\right)={\left\| {L}_{SR}-{L}_{HR} \right\|}_{1}$$

where $\:\alpha\:$ denotes the network parameters, $\:{L}_{SR}$ represents the reconstructed HR image generated by the network, and $\:{L}_{HR}$ denotes the corresponding ground truth HR image.

### Scale-aware feature adaptation (SAFA) module

In super-resolution (SR) tasks, traditional approaches typically require training dedicated networks for each specific scale factor to extract corresponding low-resolution (LR) features. This implies that each scale factor possesses its own exclusive set of network weights. However, given the demand for arbitrary-scale image super-resolution, training individual models for every potential scale factor—especially non-integer ones—is clearly impractical. Consequently, developing a single model capable of handling arbitrary scales becomes an essential necessity.

**Fig. 3:**
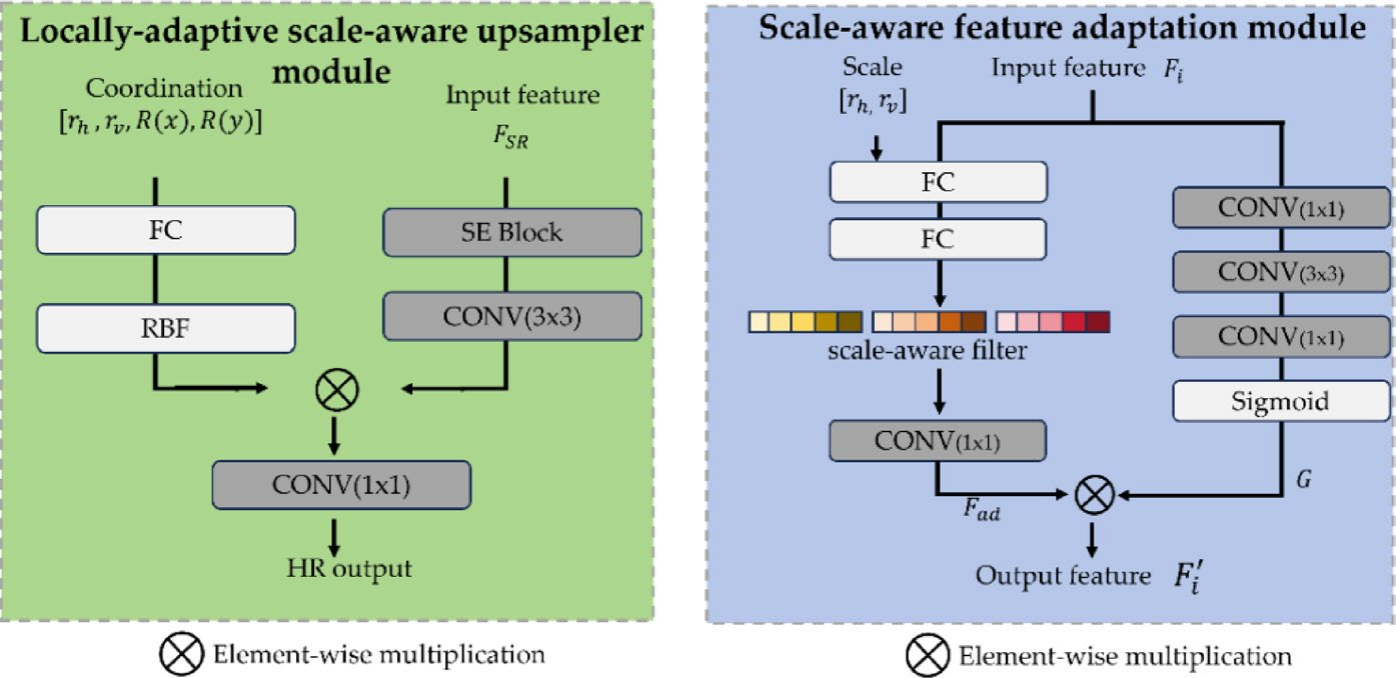
The composed of scale-aware feature adaptation and locally-adaptive scale-aware upsampler modules.

Although SR tasks for different scale factors share commonalities, their intrinsic requirements exhibit fundamental differences when the scale factors vary significantly. A critical aspect of achieving arbitrary-scale SR is extracting features relevant to the target scale. Sole reliance on the inherent feature extraction modules of the backbone network results in features lacking scale-specificity. As the scale factor increases and the number of pixels requiring reconstruction grows, feature extraction should prioritize high-frequency details; otherwise, the reconstructed output is prone to blurring. Furthermore, feature extraction depends not only on the scale factor itself but also critically on the local structural characteristics of the input image. Therefore, it is necessary to incorporate both the target scale factor information and local structural information during feature extraction. This integration generates scale-aware and locally structure-adaptive features, thereby activating more effective information for high-quality reconstruction. The scale-aware feature adaptation module, as shown in Fig. 3.

The proposed SAFA module simultaneously processes two streams. For any input feature map $\:{F}_{i}$, it is first fed into a sub-network comprising three convolutional layers followed by a sigmoid activation to produce a feature attention map $\:G$normalized to the range 0 ~ 1. Concurrently, $\:{F}_{i}$ is combined with the target scale factors $\:{r}_{h}$ and $\:{r}_{v}$ and processed by a controller network with two fully-connected (FC) layers. This generates routing weights that parameterize a scale-aware convolutional filter kernel. The dynamically generated filter is then applied to the input feature map $\:{F}_{i}$ to achieve feature adaptation. Unlike traditional convolution with fixed filters, our scale-aware convolution dynamically customizes the filter kernel conditioned on both the input features and scale information, enabling adaptive adjustment to varying scales. The adapted feature map $\:{F}_{ad}$ is obtained by processing $\:{F}_{i}$ with this scale-aware convolution. Subsequently, $\:{F}_{ad}$ is fused with the attention map $\:G$ through element-wise multiplication, expressed as:

9 $$\:{F}_{i}^{{\prime\:}}={F}_{ad}\times\:G$$

In regions with high feature similarity across different scale factors, $\:{F}_{i}$ can be directly propagated as $\:{F}_{i}^{{\prime\:}}$. Conversely, in regions exhibiting low feature similarity, $\:{F}_{i}^{{\prime\:}}$ is adaptively enhanced by incorporating $\:{F}_{ad}$. Fundamentally, $\:G$ functions as a gating mechanism that learns to guide feature adaptation at a per-pixel level. The SAFA module does not rely solely on fixed kernels; instead, it uses the scale factor together with globally aggregated context to generate channel/spatial reweighting masks that uniformly modulate the entire feature map. This introduces a globally consistent prior without requiring very deep networks or extremely large kernels, thereby alleviating the limitations of purely local convolutions in modeling large structures.

### Locally-adaptive scale-aware upsampler module

In integer-scale super-resolution (SR) tasks, conventional upsampling methods such as PixelShuffle^49^ require predefined fixed numbers of upsampling kernels for each scale factor. However, in arbitrary-scale SR scenarios, the mapping relationship between low-resolution (LR) and high-resolution (HR) pixels becomes highly scale-dependent – different scale factors correspond to distinct mapping functions. Consequently, approaches relying on predefined kernel quantities are fundamentally incompatible with arbitrary-scale reconstruction.

To address this limitation, we propose a LASU module that dynamically integrates scale factors with local features. As illustrated in Fig. 3, LASU comprises two cooperative branches. Scale-Agnostic Branch: Explores prior relationships between image feature values and spatial positions learned from training data using Squeeze-and-Excitation (SE) Blocks^50^, followed by convolutional feature refinement. This process is formulated as:

10 $$\:{F}_{da}=Conv\left({f}_{SE}\left({F}_{SR}\right)\right)$$

where $\:{F}_{da}$represents the scale-agnostic features, and $\:{f}_{SE}\left(\cdot\:\right)$ denotes the processing pipeline of the SE Blocks. The second branch—the scale-sensitive branch—performs relative positional encoding of sampling points with respect to target coordinates. This branch computes spatial geometric relationships through fully-connected layers and Radial Basis Function (RBF) activation, explicitly capturing scale-aware information. The kernels from both branches are fused to generate hybrid features $\:{F}_{hybrid}$ that simultaneously encapsulate spatial-scale relationships and semantic information:

11 $$\:{F}_{hybrid}={F}_{da}\oplus\:{F}_{SS}$$

where $\:{F}_{SS}$ contains encoded scale-spatial information. Finally, a 1 × 1 convolution adjusts channel dimensions and reconstructs the high-resolution output $\:{L}_{SR}$. The LASU module predicts content- and scale-dependent sampling offsets so that convolutions sample within an adaptive, offsettable receptive field, which is crucial for cross-scale structural alignment. Along the pyramid/multi-resolution paths, we aggregate features and use skip-wise propagation so that the low-resolution branch carrying large-range semantics/contours and the high-resolution branch carrying fine details are aligned and fused at the reconstruction head, strengthening consistent modeling of global shapes and layout^51^.

## Results

### Datasets, metrics, and experimental details

We use the DIV2K dataset 46 for training and evaluate the model on five benchmark datasets: Set5^52^, Set14^53^, BSD100^54^, Urban100^55^, Manga109, and GF1K. The GF1K dataset is composed of high-resolution optical imagery collected by the Gaofen-2 satellite constellation, including multi-spectral data with a spatial resolution of 1 m for the panchromatic band and 4 m for the multispectral bands. All images have been subjected to rigorous radiometric calibration and geometric correction.

Peak Signal-to-noise Ratio (PSNR)^56^ is used to evaluate the distortion or noise level of an image and can be expressed as follow:

12 $$\:PSNR=10{log}_{10}\left(\frac{{MAX}_{I}^{2}}{MSE}\right)=20{log}_{10}\left(\frac{{MAX}_{l}}{\sqrt{MSE}}\right)$$

13 $$\:MSE=\frac{1}{HW}\sum\:_{i=0}^{H-1}\sum\:_{j=0}^{W-1}{\left\| {I}_{SR}(i,j)-{I}_{HR}(i,j) \right\|}^{2}$$

Here, MSE represents the Mean Squared Error between the high-resolution image $\:{I}_{HR}$ and the super-resolved image $\:{I}_{SR}$. *H* and *W* denote the height and width of the image, respectively. $\:{MAX}_{l}$ denotes the maximum pixel value of the image, which is typically 255. This formula applies to grayscale image calculations. A higher value indicates better image reconstruction with less introduced noise.

The Structural Similarity Index (SSIM) assesses image similarity across three dimensions: luminance, contrast, and structure. With a maximum value of 1, higher SSIM values indicate greater similarity between images. The specific calculation formula is shown in Eq. 14.

14 $$\:SSIM({I}_{SR},{I}_{HR})=\frac{(2{\gamma\:}_{{I}_{SR}}{\gamma\:}_{{I}_{HR}}+{C}_{1})(2{\sigma\:}_{{I}_{SR}{I}_{HR}}+{C}_{2})}{({\gamma\:}_{{I}_{SR}}^{2}+{\gamma\:}_{{I}_{HR}}^{2}+{C}_{1})({\sigma\:}_{{I}_{SR}}^{2}+{\sigma\:}_{{I}_{HR}}^{2}+{C}_{2})}$$

In practical implementations, a sliding window protocol is typically employed to partition the image into sub-regions. The SSIM value of each sub-region is computed individually, and the average SSIM value across all sub-regions serves as the composite structural similarity metric between the original high-resolution (HR) image and the reconstructed image.

During the training phase, we adopt specific data generation strategies for different types of scaling factors. For symmetric scaling factors, we generate corresponding low-resolution (LR) training images for each scale factor with a step size of 0.1. For asymmetric scaling factors, we generate LR training images by independently varying the vertical and horizontal scaling factors with a step size of 0.5. From the generated images, we randomly crop image patches of size 50 × 50 within each training batch as input to the network. Model optimization is performed using the Adam algorithm^57^, with hyperparameters set to β₁ = 0.9 and β₂ = 0.999. The initial learning rate is set to 1 × 10⁻⁴ and is halved every 20 epochs. The entire training process lasts for 120 epochs with a batch size of 8. The model is implemented and trained using the PyTorch framework and executed on two NVIDIA GeForce GTX 4070 Ti GPUs.

### Comparison method

In this section, we compare our method with MetaSR, ArbSR, LIIF^58^, LTE^59^, and CLIT^60^ on super-resolution (SR) tasks with symmetric scaling factors, including both integer and non-integer scale factors. MetaSR introduces a pioneering meta-upsampling module for arbitrary scale upsampling. ArbSR is the first method to achieve reconstruction with asymmetric scaling factors in arbitrary-scale SR. LIIF and LTE are state-of-the-art (SOTA) methods in arbitrary-scale SR based on implicit functions. CLIT, a Transformer-based model, is also a SOTA method for arbitrary-scale SR.

### Results on SR with symmetric scaling factors

The scale factors in our experiments were carefully chosen rather than arbitrarily selected. The main purpose of this study is to demonstrate the proposed network’s ability to perform arbitrary-scale super-resolution, covering both integer and non-integer magnifications. Limiting the evaluation to standard ×2, ×3, and ×4 settings would not fully reflect this flexibility. Moreover, different datasets have distinct native resolutions and scene characteristics. For example, Urban100 contains high-resolution urban details, and we follow the common practice of testing ×2, ×3, and ×4 for fair comparison with previous works. In contrast, BSD100 images are smaller and contain fewer high-frequency details; thus, we evaluate fractional scales such as ×1.4, ×3.2, and ×3.5 to simulate practical cases and demonstrate the model’s robustness to non-integer magnifications. Overall, the selected scale factors correspond to the practical resolution gaps of each dataset and reflect typical real-world super-resolution requirements, which primarily fall between ×1.4 and ×4.

Tables 1 and 2 present the average PSNR and SSIM results of different methods on the Set5, Set14, BSD100, Urban100, Manga109 and GF1K datasets. the best results are shown in bold. As observed from the tables, our method consistently achieves the best performance for integer scaling factors. Compared to the CLIT method, our model improves the average PSNR and SSIM by 0.28 dB and 0.008 on Urban100 and Manga109, respectively. For non-integer scaling factors, our method significantly outperforms all other approaches. For example, on Set14, our network achieves ×2.8 SR with a PSNR of 31.34 dB and an SSIM of 0.8655, and demonstrates even more notable advantages for ×3.2 SR.

**Table 1:** Result of average PSNR for different methods.

Dataset×scale	Bicubic	MetaSR	ArbSR	LIIF	LTE	CLIT	Ours
Urban100_×2	26.88	33.12	33.14	32.87	33.38	33.13	**33.52**
Urban100_×3	24.46	28.96	28.98	28.82	29.21	29.04	**29.39**
Urban100_×4	23.14	26.71	26.68	26.68	26.94	26.91	**27.01**
Managa109_×2	30.80	39.32	39.37	39.21	39.52	39.49	**39.66**
Managa109_×3	26.95	34.40	34.55	34.14	34.77	34.63	**34.90**
Managa109_×4	24.89	31.33	31.36	31.20	**31.67**	31.58	30.72
Set5_×2	33.66	38.22	38.24	38.30	38.30	38.26	**38.33**
Set5_×2.4	32.41	36.51	36.59	36.47	36.66	36.06	**36.71**
Set5_×3.25	29.21	33.98	34.03	34.12	34.16	34.17	**34.23**
Set14_×1.5	32.87	37.51	37.53	37.45	34.25	37.68	**37.79**
Set14_×2.8	27.84	31.00	31.01	30.97	31.28	31.30	**31.34**
Set14_×3.2	26.98	28.86	28.93	28.90	30.32	30.22	**30.35**
BSD100_×1.4	32.95	36.88	36.87	36.91	36.92	36.94	**39.99**
BSD100_×3.2	26.91	28.86	28.93	28.90	29.00	28.95	**29.03**
BSD100_×3.5	26.32	28.27	28.33	28.34	28.24	28.40	**28.47**
GF1K_×1.5	32.53	38.73	36.94	37.02	34.55	37.89	**38.81**
GF1K_×2.5	30.45	33.66	30.51	31.01	30.96	32.08	**33.69**
GF1K_×3.4	24.11	28.99	29.05	28.61	29.98	30.10	**30.12**

**Table 2:** Result of average SSIM for different methods.

Dataset×scale	Bicubic	MetaSR	ArbSR	LIIF	LTE	CLIT	Ours
Urban100_×2	0.8410	0.9374	0.9367	0.9352	0.9388	0.9363	**0.9410**
Urban100_×3	0.7370	0.8665	0.8674	0.8663	0.8730	0.8706	**0.8742**
Urban100_×4	0.6585	0.8083	0.8047	0.8040	0.8100	0.8094	**0.9129**
Managa109_×2	0.9339	0.9782	0.9785	0.9772	0.9791	0.9789	**0.9799**
Managa109_×3	0.8556	0.9488	0.9501	0.9478	0.9508	0.9503	**0.9554**
Managa109_×4	0.7866	0.9180	0.9196	0.9170	0.9115	0.9203	**0.9208**
Set5_×2	0.9299	0.9612	0.9613	0.9615	0.9615	0.9614	**0.9615**
Set5_×2.4	0.9072	0.9482	0.9487	0.9479	**0.9504**	0.9496	0.9500
Set5_×3.25	0.8532	0.9216	0.9225	0.9212	0.9242	0.9238	**0.9247**
Set14_×1.5	0.9268	0.9575	0.9581	0.9578	0.9231	0.9588	**0.9610**
Set14_×2.8	0.7932	0.8616	0.8622	0.8615	0.8647	0.8652	**0.8655**
Set14_×3.2	0.7595	0.7932	0.7958	0.8321	0.8351	0.8367	**0.8372**
BSD100_×1.4	0.9031	0.9639	0.9644	0.9632	0.9645	0.9639	**0.9646**
BSD100_×3.2	0.7235	0.7933	0.7958	0.7937	0.7969	0.7969	**0.7979**
BSD100_×3.5	0.6990	0.7944	0.7785	0.7764	0.7799	0.7794	**0.7818**
GF1K_×1.5	0.9162	0.9452	0.9463	0.9481	0.9184	0.9485	**0.9501**
GF1K_×2.5	0.7830	0.8591	0.8567	0.8573	0.8537	0.8591	**0.8597**
GF1K_×3.4	0.7532	0.7921	0.7910	0.8316	**0.8361**	0.8314	0.8344

**Fig. 4:**
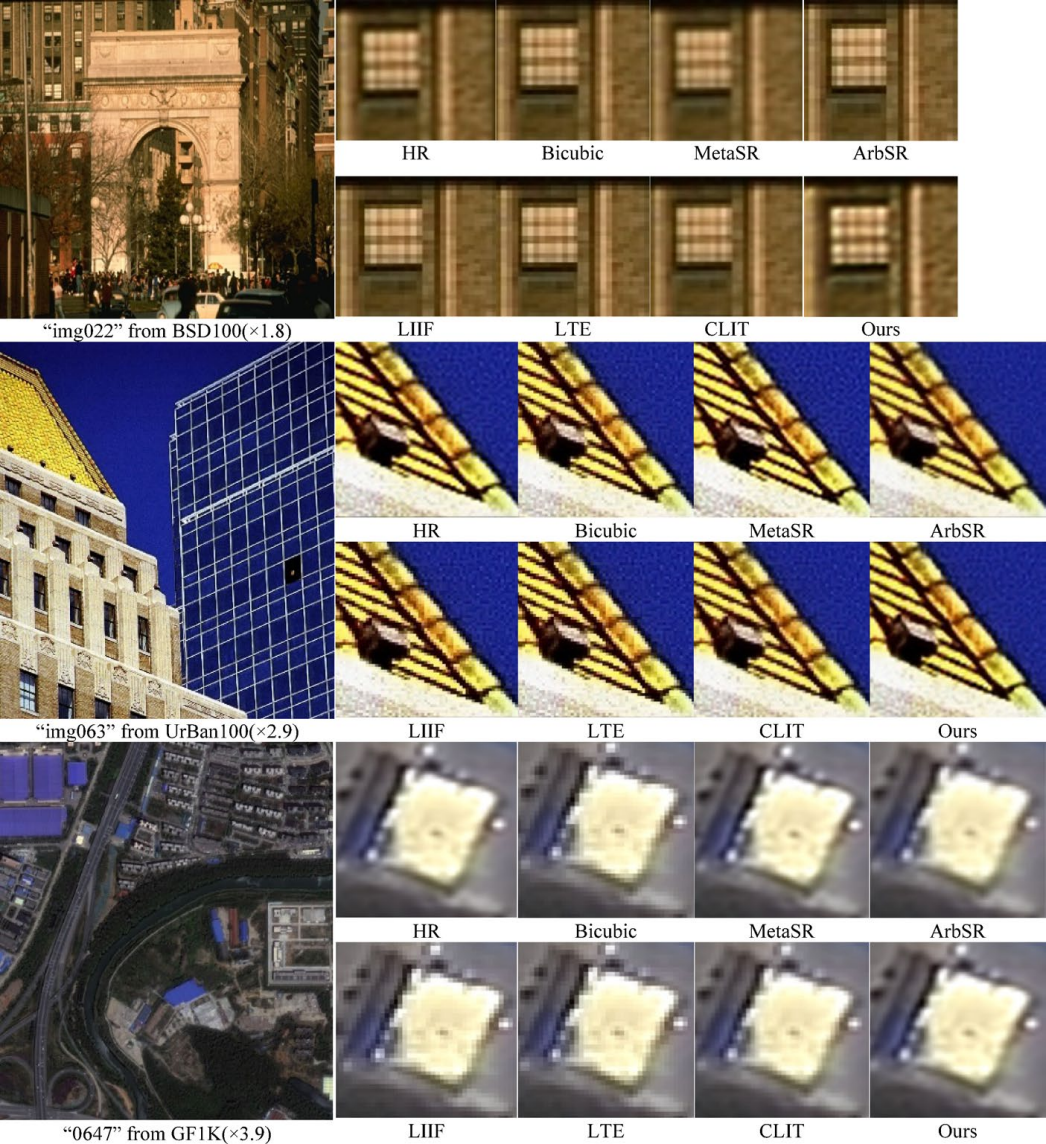
Results of different SR algorithms under multiple symmetric scaling factors. Images are from the BSD100, Urban100 and GF1K datasets.

In the field of remote sensing imagery, factors such as sensor noise, optical distortion, and environmental interference can adversely affect image quality, making it challenging to acquire high-resolution remote sensing images. Unlike natural optical images, satellite remote sensing images exhibit smaller spatial resolution, meaning each pixel potentially represents more ground information. Consequently, there is a heightened need for arbitrary-scale super-resolution capability to enhance their utility for downstream applications. As demonstrated in Tables 1 and 2 on the GF1K dataset, our method significantly enhances the clarity of image texture details and outperforms other methods more markedly on remote sensing image indicators compared to natural optical image benchmarks. This demonstrates its strong applicability and potential for serving the satellite remote sensing domain.

**Fig. 5:**
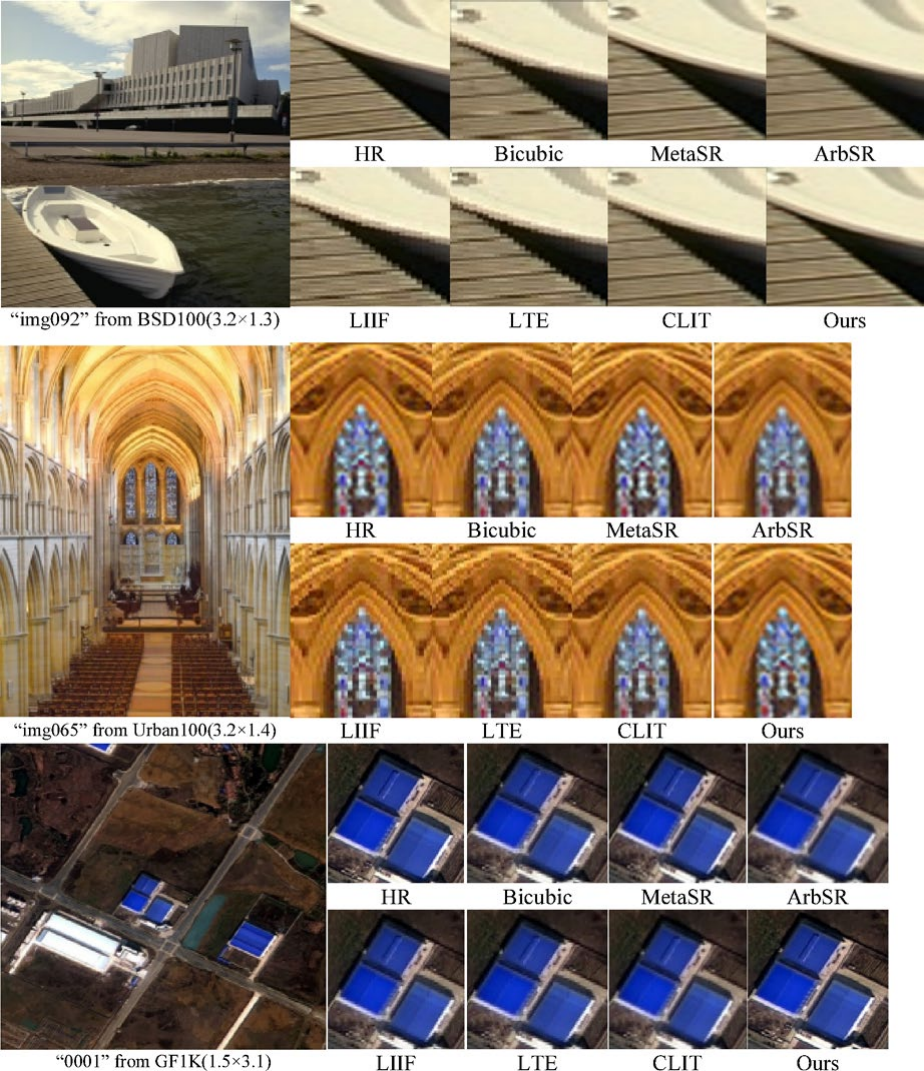
Results of different SR algorithms under various asymmetric scaling factors. Images are from the BSD100, Urban100 and GF1K datasets.

Figure 4 shows the visual comparison results of different SR algorithms at multiple symmetric scaling factors. It can be observed that most competing methods produce blurry outputs. Specifically, Fig. 4 presents the ×1.8 SR result of image img022 from the BSD100 dataset. Compared to other methods, our approach is able to more clearly restore the fine texture details of dense line patterns. This improvement is attributed to the more effective multi-level feature extraction and fusion strategies employed in our network. As a result, our model demonstrates a significant advantage in recovering cross-hatch structures, repetitive textures, and other high-frequency dense details. Similarly, in Fig. 5, only our model is able to accurately reconstruct the triangular patterns in the image, thereby achieving better perceptual quality and fewer artifacts. This benefit arises from the integration of scale and local feature information into the upsampling process in our model, allowing the upsampling kernels to perform geometric transformations based on local features. Consequently, the reconstructed outputs are better adapted to the image content.

### Results on SR with asymmetric scaling factors

**Table 3:** Result of average PSNR and SSIM for different methods.

Method	Metrics	Set5	Set14	BSD100	Urban100	Managa109	GF1K
		$\:\frac{\times\:1.5}{\times\:4}$	$\:\frac{\times\:3.5}{\times\:2}$	$\:\frac{\times\:3.5}{\times\:1.5}$	$\:\frac{\times\:1.6}{\times\:3.9}$	$\:\frac{\times\:2.8}{\times\:3.2}$	$\:\frac{\times\:3.1}{\times\:2.9}$
Bicubic	PSNR	30.01	27.88	27.91	24.91	26.47	27.94
	SSIM	0.8640	0.7937	0.7737	0.7542	0.8411	0.8053
MetaSR	PSNR	34.20	31.05	30.09	29.03	33.63	33.23
	SSIM	0.9235	0.8638	0.8402	0.8687	0.9520	0.9152
ArbSR	PSNR	34.37	31.27	30.21	29.36	33.81	33.65
	SSIM	0.9246	0.8687	0.8414	0.8731	0.9524	0.9196
LIIF	PSNR	34.30	31.16	30.22	29.28	33.74	33.16
	SSIM	0.9244	0.8673	0.8408	0.8714	0.9519	0.9167
LTE	PSNR	34.53	31.52	30.27	29.58	34.05	**34.08**
	SSIM	0.9260	0.8703	0.8422	0.8754	0.9530	0.9348
CLIT	PSNR	34.47	31.44	30.25	29.54	33.96	33.85
	SSIM	0.9252	0.8701	0.8421	0.8751	0.9530	0.9389
Ours	PSNR	**34.66**	**31.62**	**30.35**	**29.75**	**34.16**	34.01
	SSIM	**0.9274**	**0.8726**	**0.8488**	**0.8774**	**0.9541**	**0.9493**

Table 3 presents the average PSNR and SSIM results of different methods on the Set5, Set14, BSD100, Urban100, Manga109 and GF1K datasets. Since methods like MetaSR and LIIF do not support asymmetric scaling, their performance is affected by the limitations of downsampling operations. Our model explicitly considers the different impacts of horizontal and vertical scaling on image super-resolution (SR) during training and integrates low-resolution (LR) images with asymmetric scaling factors. This design leads to significant performance improvements.

For example, on the asymmetric ×2.8/×3.2 SR task in the Manga109 dataset, our method achieves clearly superior results (PSNR: 34.16 dB; SSIM: 0.9541) compared to competing methods. This improvement is primarily attributed to the model’s ability to effectively leverage multiple sources of information, such as local features and scaling factors, allowing it to restore image details more accurately according to content.

Figure 5 presents a visual comparison of different super-resolution (SR) algorithms under various asymmetric scaling factors. It can be observed that most SR reconstruction results suffer from texture blurring, structural distortion, and significant loss of edge details. In contrast, our method accurately perceives local line orientations and generates fine textures, achieving superior visual quality. Notably, Fig. 5 further demonstrates that existing methods generally struggle to accurately reconstruct repetitive texture structures, often resulting in visible distortion and artifacts. Only our method is able to clearly recover densely packed line features. This advantage is attributed to the model’s effective utilization of local neighborhood features—by enhancing attention to relevant regions, it significantly improves the perception of edges and high-frequency details. This enhanced capability effectively suppresses reconstruction bias and preserves the original dense and repetitive structures, directly contributing to the outstanding visual performance of our model in SR tasks with asymmetric scaling factors.

### Ablation study

**Table 4:** Results of PSNR Obtained Using Different Modules.

SAFA module	Skip connection	LASU module	$\:\times\:$2	$\:\times\:$3.25	$\:\times\:4.1$	$\:\frac{\times\:1.5}{\times\:4}$	$\:\frac{\times\:3.4}{\times\:2.3}$
×	×	×	38.08	33.97	31.05	34.36	35.66
×	×	√	38.13	34.01	31.27	34.47	35.67
√	×	√	38.24	34.12	31.39	34.59	35.78
√	√	√	**38.33**	**34.23**	**31.51**	**34.66**	**35.83**

We conducted a comprehensive ablation study on the Set5 dataset to evaluate the individual and combined contributions of the key components in our network. The Peak Signal-to-Noise Ratio (PSNR) results for various scaling factors (×2, ×3.25, ×4.1, ×1.5/×4 and ×3.4/2.3) under different component configurations are summarized in Table 4.

The baseline model, lacking all three components, achieves PSNR values of 38.08 dB (×2), 33.97 dB (×3.25), 31.05 dB (×4.1), 34.36 dB (×1.5/×4) and 35.66 dB (×3.4/2.3). Introducing only the Dynamic Upsampling module yields consistent improvements across all scales, increasing the PSNR to 38.13 dB (×2), 34.01 dB (×3.25), 31.27 dB (×4.1), 34.47 dB (×1.5/×4) and 35.67 dB (×3.4/2.3), demonstrating its inherent effectiveness in enhancing reconstruction quality. Finally, incorporating all three components produces the best results: 38.33 dB (×2), 34.23 dB (×3.25), 31.51 dB (×4.1) 34.66 dB (×1.5/×4) and 35.83 dB (×3.4/2.3). These results unequivocally demonstrate the positive contribution of each component and, more importantly, their synergistic effect when combined, leading to state-of-the-art performance across diverse magnification factors.

### Investigation of model complexity and inference time

**Fig. 6:**
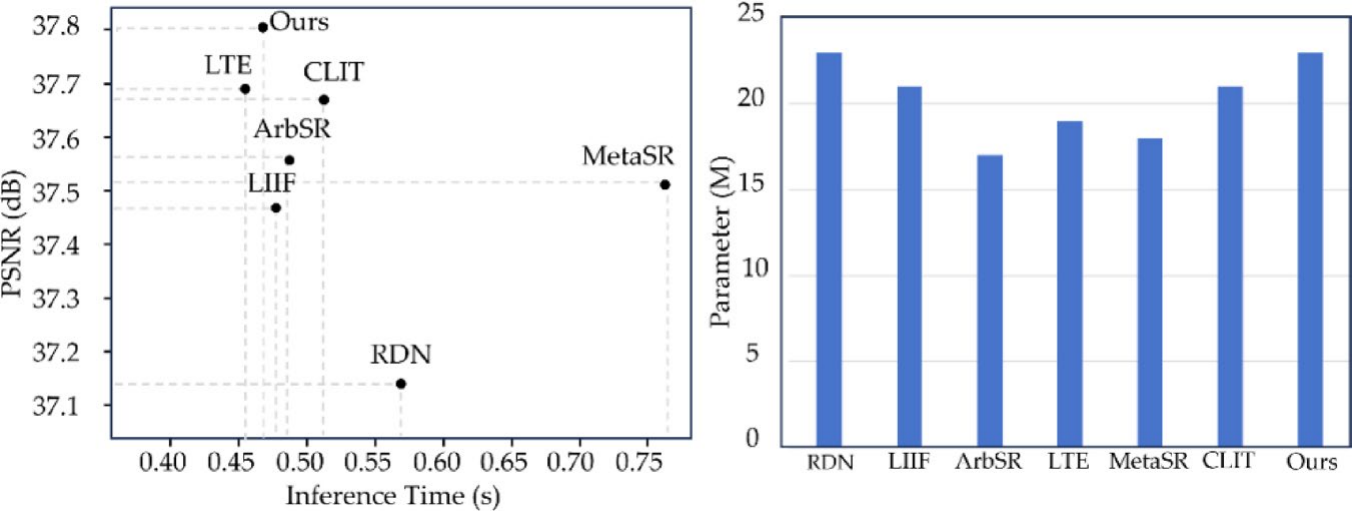
Inference Time, PSNR, and Model Size of Various Algorithms.

In this section, we evaluate the model’s computational complexity in terms of inference time and the number of parameters. Figure 6 compares the inference time (in seconds) and PSNR (in dB), as well as the number of parameters (in millions) and PSNR across different methods.

As shown in the Fig. 6, our model has a lower average inference time than MetaSR and is slightly higher than ArbSR and LTE, effectively maintaining competitive speed. In terms of parameters, our network incorporates two sets of plug-and-play modules, resulting in an increased parameter count compared to ArbSR. However, this slight increase in parameters does not significantly affect the inference time, while delivering a notable improvement in PSNR. Overall, our method demonstrates a clear advantage in computational efficiency.

## Conclusion

Single image super-resolution (SISR) has evolved significantly in recent years, progressing from traditional approaches to deep learning-based methods. However, existing models still face two major challenges: the loss of scale-awareness during arbitrary-scale feature extraction and limitations in structural modeling, as well as deficiencies in upsampling mechanisms. To address these issues, we propose a novel arbitrary-scale super-resolution framework based on Content-aware Dynamic Adaptive Sampling and Scale-aware Representation, named CDASSR-Net. First, a scale-aware feature adaptation module dynamically responds to scaling factor changes by adjusting filter parameters accordingly. Second, we introduce a cross-scale feature fusion mechanism, which efficiently aggregates multi-scale features via skip connections to prevent detail loss in deep networks. Finally, by leveraging scale information to dynamically generate convolution kernels, our model supports generalizable reconstruction across arbitrary resolutions.

Experiments on multiple benchmark datasets demonstrate that embedding our proposed modules into fixed-scale SR networks enables superior reconstruction performance at non-integer and asymmetric scales, while fully preserving state-of-the-art results at integer scales.

For future work, we plan to further improve the efficiency of arbitrary-scale super-resolution models, explore extensions to video super-resolution, and investigate adaptive scale-aware modules for different imaging modalities, such as medical or satellite imagery. Additionally, we aim to study more effective mechanisms for preserving high-frequency details under extreme or non-integer scale factors.

## Data Availability

The datasets generated or analysed during the current study are available in the repository. DIV2K: https://data.vision.ee.ethz.ch/cvl/DIV2K/. Set5, Set14, BSD100, Urban100, and Manga109: https://pan.baidu.com/s/1qeftNHrWSjLxfhJCjfqNyw?pwd=9ag4. GF1K: https://pan.baidu.com/share/init?surl=NeFj2gnAHuq0tKdZW_bqoA?pwd=isku.
